# The association between dietary carbohydrate intake and the risk of hyperlipidemia among reproductive-aged women in the US: A cross-sectional study

**DOI:** 10.1371/journal.pone.0310184

**Published:** 2024-10-16

**Authors:** Minli Zhao, Qiuping Zhang, Yuan Lin, Danwei Zhang, Hua Cao

**Affiliations:** 1 Fujian Children’s Hospital (Fujian Branch of Shanghai Children’s Medical Center), College of Clinical Medicine for Obstetrics & Gynecology and Pediatrics, Fujian Medical University, Fuzhou, China; 2 Shengli Clinical Medical College of Fujian Medical University, Fuzhou, China; Xi’an Jiaotong University, CHINA

## Abstract

**Background:**

The association between dietary carbohydrate intake and hyperlipidemia remained incompletely understood. This study aimed to explore the association between dietary carbohydrate intake and the risk of hyperlipidemia among reproductive-aged women in the US.

**Methods:**

The study utilized data from the National Health and Nutrition Examination Survey (NHANES) conducted from 2005 to 2020. Dietary intake information was assessed via interviews using 24-hour dietary recall interviews, and hyperlipidemia diagnosis adhered to the National Cholesterol Education Program guidelines. Univariate and multivariate logistic regression analyses, along with restricted cubic splines (RCS) and stratified analyses, were conducted to investigate the association between dietary carbohydrate intake and the risk of hyperlipidemia.

**Results:**

A total of 6,791 women of reproductive age, with a mean age of 34.87 (±8.57) years, were included in the final analysis. In the multivariate logistic regression model adjusting for covariates, a higher percentage of energy from carbohydrate was positively correlated with the risk of hyperlipidemia (adjusted odds ratio (AOR): 1.014, 95% CI: 1.004–1.024). Analyzing the percentage of energy from carbohydrate as a categorical variable, compared to the lowest quartile, the third quartile (AOR: 1.263, 95% CI: 1.031–1.546) and the highest quartile (AOR: 1.411, 95% CI: 1.083–1.839) were associated with increased hyperlipidemia risk. Additionally, a linear relationship (*P* for nonlinearity = 0.088) existed between the percentage of energy from carbohydrate and the risk of hyperlipidemia, with an inflection point identified at 49.64.

**Conclusions:**

This study found that elevated dietary carbohydrate intake was associated with an increased the risk of hyperlipidemia in reproductive-aged women. These findings implied that reproductive-aged women should pay closer attention to reducing their carbohydrate intake.

## 1. Introduction

Hyperlipidemia, a prevalent metabolic disorder, was characterized by elevated lipid levels in the blood, particularly total cholesterol (TC) and triglycerides (TG) [1]. According to a WHO estimate, approximately 39% of adults aged ≥25 years globally had elevated TC levels [2], while in the US from 2007 to 2018, about 11.5% of adults aged ≥18 years had TC levels ≥240 mg/dL, and 10.4% had TG levels ≥200 mg/dL [3]. Currently, hyperlipidemia has become a global public health issue, imposing a significant burden on both socio-economic development and public health [2].

Hyperlipidemia is not only associated with disturbances in the levels of important adipokines [4], cardiovascular diseases such as atherosclerosis [5], metabolic diseases such as hypothyroidism [6], mental disorders such as depression [7], and other chronic diseases such as non-alcoholic fatty liver disease and kidney failure [8, 9], but also directly impacts reproduction health and pregnancy outcomes. Hyperlipidemia may disrupt the normal functioning of the reproductive system in reproductive-aged women, resulting in menstrual disorders and polycystic ovary syndrome, which may ultimately cause fertility [10]. Furthermore, hyperlipidemia significantly increase the risk of maternal gestational complications such as preeclampsia or eclampsia, and gestational diabetes mellitus [11–13]. It may also lead to adverse outcomes for the offspring, including prematurity, macrosomia and birth defects [14, 15]. Therefore, timely identification of modifiable risk factors for hyperlipidemia is highly significant in preventing and reducing its incidence among women of reproductive age.

Diet plays an important role in the primary prevention of chronic diseases and in lipid management [16]. In the majority of global populations, carbohydrate serve as the primary energy source, contributing 50% or more of daily energy intake, while fat and protein make up a smaller proportion [17]. In recent years, reducing carbohydrate intake has gained popularity as a strategy for weight loss and weight management [18]. A high-carbohydrate intake of carbohydrate could potentially have adverse effects on the body. For instance, several studies have demonstrated an association between high- dietary carbohydrate intake and an increased risk of metabolic syndrome, cardiovascular diseases and mortality [19–21]. To the best of our knowledge, the association between dietary carbohydrate intake and the risk of hyperlipidemia among reproductive-aged women has not been evaluated.

This study aimed to investigate the association between the dietary carbohydrate intake and the risk of hyperlipidemia by analyzing data from the National Health and Nutrition Examination Survey (NHANES), a cross-sectional survey conducted in the US. We hypothesized that an increased dietary carbohydrate intake might elevate the risk of hyperlipidemia among reproductive-aged women.

## 2. Methods

### 2.1 Study design and population

In this study, data were drawn from NHANES, a cross-sectional survey conducted to assess the health and nutritional status of the general US population, carried out in two-year cycles. The protocol was approved by the Institutional Review Board of National Center for Health Statistics, and all participants provided their written informed consent. More information about NHANES can be found at https://www.cdc.gov/nchs/nhanes/index.htm. All methods utilized in this study were conducted in strict adherence to the principles outlined in the Declaration of Helsinki.

**Fig 1:**
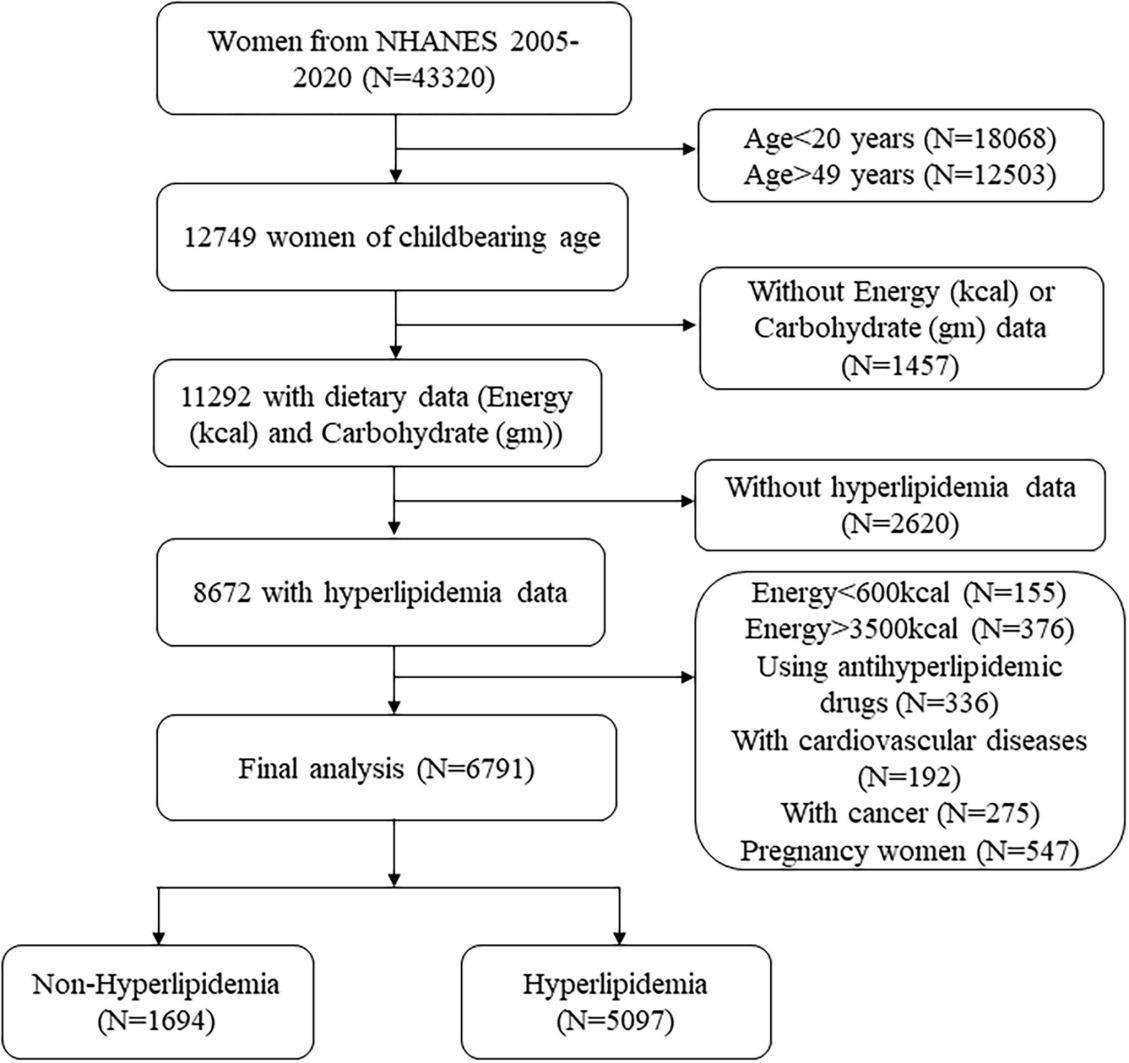
The flow chart of participant selection.

From 2005 to 2020, a total of 43,320 women participated in the NHANES survey. This study included women of reproductive age as 20–49 years (n = 12749). Subsequently, 1,457 women were excluded due to missing total energy (kcal) and/or carbohydrate (gm) data, and 2,620 women were excluded due to missing hyperlipidemia data. Finally, 1881 women were excluded due to unreliable dietary intake (defined as daily calorie intake levels below 600 kcal or above 3500 kcal), the use of antihyperlipidemic drugs, a diagnosis of cardiovascular diseases (CVDs) or cancer, or pregnancy. A total of 6,791 reproductive-aged women were ultimately included in the final analysis. Fig 1 provided a detailed illustration of the selection process for the study population.

### 2.2 Assessment of percent energy from carbohydrate

Each participant’s dietary intake in the NHANES dataset was assessed through a 24-hour dietary recall interview conducted by a trained interviewer. The recall data encompassed all foods and beverages consumed by participants within a 24-hour period. This study included only the data from the first 24-hour dietary recall due to the smaller sample size of the data obtained from the second recall, which was conducted via telephone interview. This study applied standard conversion factors (4 kcal/g for carbohydrate and protein, and 9 kcal/g for fat intake) to convert grams to kilocalories (kcal) [22]. The NHNAES dataset already included total energy intake in the data file. The exposure variable used in this study was the percentage of energy from carbohydrate, calculated as follows:

$$Percent\;energy\;from\;carbohydrate=\frac{Carbohydrate\;\left(g\right)*4\;kcal/g}{Total\;energy}*100$$

### 2.3 Assessment of hyperlipidemia

Hyperlipidemia status was determined based on the adult National Cholesterol Education Program guidelines. These guidelines define hyperlipidemia as meeting any of the following criteria: TC≥ 200 mg/dL, TG ≥ 150 mg/dL, high-density lipoprotein (HDL) ≤ 50 mg/dL (all participants in this study were women), or low-density lipoprotein (LDL) ≥ 130 mg/dL [23]. Additionally, women who reported the use of cholesterol-lowering medications were also considered to have hyperlipidemia [24, 25].

### 2.4 Other covariables

To assess the impact of potential confounding factors, this study included several covariates: age, body mass index (BMI), marital status, race/ethnicity, education level, poverty income ratio (PIR), drinking status, smoking status, diabetes mellitus, hypertension, total energy intake, percentage of energy from protein, and percentage of energy from fat. The age of the women considered in this study was their age at the time of screening. BMI was calculated as weight (kg) divided by height (m^2^) and classified as underweight (<20 kg/m2), normal (≥20 to <25 kg/m2), overweight (≥25 to <30 kg/m2), and obese (≥30 kg/m2) [26]. Race/ethnicity was divided into Mexican American, non-Hispanic Black, non-Hispanic White, other Hispanic, and other race/ethnicity [27, 28]. Marital status was classified as living alone (separated, widowed, never married, or divorced) and married (married or living with partners) [27, 29]. Education level was graded as lower than high school (including 12th grade with no diploma), high school (including equivalent), and above high school [30]. The PIR was defined as low (≤1.5), middle (1.5–3.5), or high (>3.5) [25, 27]. Drinking status was classified as never (reported drinking <12 drinks), former (more than 12 drinks in lifetime but not in the past year), heavy (≥3 drinks per day), moderate (≥2 drinks per day), and mild (did not meet the all above criteria) [31]. Smoking status was classified as never (smoked <100 cigarettes in lifetime), former (smoked >100 cigarettes in lifetime but currently did not smoke at all), and current (smoked >100 cigarettes in lifetime and currently smoked some days or every day) [31]. Hypertension was defined as an average blood pressure >140 mmHg systolic and/or 90 mmHg diastolic, report of hypertension diagnosed by a physician, or taking hypertension medicine [24]. Diabetes mellitus was defined as reporting a diabetes mellitus diagnosis, glycohemoglobin HbA1c (%) > 6.5, or fasting glucose (mmol/L) ≥ 7.0, random blood glucose (mmol/L) ≥ 11.1, 2h OGTT blood glucose (mmol/L) ≥ 11.1, or use of diabetes mellitus medication or insulin [32]. This study computed the metabolic equivalent (MET) minutes for these activities using the transformation recommended by NHANES. Total physical activity was calculated as the sum of all MET minutes per week across all questions related to physical activity. This methodology is widely adopted due to its capability to accurately assess the frequency and intensity of an individual’s physical activity throughout the week [33]. Missing values were imputed using multiple imputation.

### 2.5 Statistical analysis

Participants were categorized into quartiles based on their percentage of energy from carbohydrate, ranging from the lowest (Q1) to the highest (Q4). Continuous variables were expressed as mean (± standard deviation) and analyzed using a t-test for two groups and one-way ANOVA for more than two groups. Categorical variables were presented as percentages (%) and analyzed using the chi-square test. Univariate and multivariate logistic regression models were employed to assess the association between the percentage of energy from carbohydrate and the risk of hyperlipidemia. Model 1 was unadjusted, Model 2 was adjusted for age, BMI, race, marital status, education level, PIR, drinking status, smoking status, diabetes mellitus, hypertension, and physical activity, while Model 3 additionally adjusted for total energy intake, percentage of energy from protein, and percentage of energy from fat. Restricted cubic spline (RCS) analysis was employed to explore potential nonlinear relationships between the percentage of energy from carbohydrate and the risk of hyperlipidemia. This study conducted a subgroup analysis stratified by various factors, including age, BMI, marital status, ethnicity, educational level, PIR, drinking status, smoking status, diabetes mellitus, and hypertension, to assess the association between the percentage of energy from carbohydrate and the risk of hyperlipidemia. In sensitivity analysis, this study examined the association between the percentage of energy from carbohydrate and hyperlipidemia after excluding individuals with diabetes mellitus. All analyses were conducted using R 4.2.2 and SPSS 27, with statistical significance set at *P* < 0.05.

## 3. Results

### 3.1 Demographic characteristics

This study included 6,791 woman aged 20–49 years, with an average age of 34.87 (±8.57) years and an average BMI of 29.81 (±8.04) kg/m^2^. The mean daily total energy intake was 1852.85 (±640.20) kcal/day, with an average percentage of energy from carbohydrate at 49.61 (±11.18). Table 1 displayed the differences in clinical characteristics between reproductive-aged women with hyperlipidemia and those without hyperlipidemia. Compared to reproductive-aged women without hyperlipidemia, those with hyperlipidemia were older, had higher BMI levels, were more likely to be married, non-Hispanic white, had education levels less than high school, had a low PIR level, have a mild drinking status, were current smokers, and had diabetes mellitus or hypertension. Additionally, they had higher levels of percent energy from carbohydrate and lower levels of percent energy from fat and protein. However, there were no differences in total energy intake and physical activity between two groups. Table 2 presented the clinical characteristics of the participants based on the quartiles of percent energy from carbohydrate. Women in quartile 4 tended to be non-Hispanic white, possessed education levels above high school, had a low PIR level, mild drinking status, were non-smokers, had hyperlipidemia, exhibited lower levels of total energy and physical activity, and exhibited higher levels of percent energy from carbohydrate along with lower levels of percent energy from fat and protein. There was a significant difference among the percent energy from carbohydrate quartiles in terms of race, educational level, PIR, drinking status, smoking status, physical activity, hyperlipidemia, total energy, percent energy from carbohydrate, percent energy from fat, and percent energy from protein (all *P* < 0.05).

**Table 1:** Characteristics of the study population based on hyperlipidemia.

Characteristic	All participants	Hyperlipidemia	Non-hyperlipidemia	*P*
**Number of participants**	6791	5097	1694	
**Age, mean (SD)**	34.87 (±8.57)	35.51(±8.54)	32.94 (±8.36)	< 0.001
as categorical variable, n (%)				< 0.001
20–29	2096(30.86)	1448(28.41)	648(38.25)	
30–39	2308(33.99)	1701(33.37)	607(35.83)	
40–49	2387(35.15)	1948(38.22)	439(25.91)	
**BMI, mean (SD)**	29.81 (±8.04)	30.91 (±8.05)	26.50(±7.03)	< 0.001
as categorical variable, n (%)				< 0.001
underweight	412(6.07)	192(3.77)	220(12.99)	
normal weight	1711(25.20)	1052(20.64)	659(38.90)	
overweight	1790(26.36)	1391(27.29)	399(23.55)	
obese	2878(42.38)	2462(48.30)	416(24.56)	
**Marital status, n (%)**				< 0.001
Single	2731(40.21)	1990(39.04)	741(43.74)	
Married	4060(59.79)	3107(60.96)	953(56.25)	
**Race/ethnicity, n (%)**				< 0.001
Mexican American	1296(19.08)	1039(20.38)	257(15.17)	
Other Hispanic	686(10.10)	529(10.38)	157(9.27)	
Non-Hispanic white	2440(35.93)	1850(36.30)	590(34.83)	
Non-Hispanic black	1424(20.97)	1007(19.76)	417(24.62)	
Other	945(13.92)	672(13.18)	273(16.12)	
**Educational level, n (%)**				< 0.001
Less than high school	1306(19.23)	1067(20.93)	239(14.11)	
High school	1352(19.91)	1052(20.64)	300(17.71)	
Above high school	4133(60.86)	2978(58.43)	1155(68.18)	
**PIR, n (%)**				0.001
Low	2829(41.66)	2185(42.87)	644(38.02)	
Middle	2066(30.42)	1533(30.08)	533(31.46)	
High	1896(27.92)	1379(27.06)	517(30.52)	
**Drinking status, n (%)**				0.027
Never	1050(15.46)	787(15.44)	263(15.53)	
Former	499(7.35)	390(7.65)	109(6.43)	
Mild	2021(29.76)	1546(30.33)	475(28.04)	
Moderate	1604(23.62)	1161(22.78)	443(26.15)	
Heavy	1617(23.81)	1213(23.80)	404(23.85)	
**Smoking status, n (%)**				< 0.001
Never	4817(70.93)	3507(68.81)	1310(77.33)	
Former	706(10.40)	538(10.56)	168(9.92)	
Current	1268(18.67)	1052(20.64)	216(12.75)	
**Diabetes mellitus, n (%)**				< 0.001
Yes	406(5.98)	365(7.16)	41(2.42)	
No	6385(94.02)	4732(92.84)	1653(97.58)	
**Hypertension, n (%)**				< 0.001
Yes	979(14.42)	844(16.56)	135(7.97)	
No	5812(85.58)	4253(83.44)	1559(92.03)	
**Physical activity, MET-min/week**	1986.00(727.00, 5206.00)	1927.00(727.00, 5255.00)	2166.00(847.00, 5190.25)	0.051
**Total energy, mean (±SD)**	1852.85(±640.20)	1843.12 (±636.18)	1882.13 (±651.47)	0.095
**Protein (g), mean (±SD)**	70.06 (±30.27)	69.26(±29.77)	72.48 (±31.63)	0.002
**% of energy, mean (±SD)**	15.41 (±5.09)	15.32 (±5.07)	15.67 (±5.16)	0.041
**Fat (g), mean (±SD)**	71.46 (±33.58)	70.65 (±33.24)	73.93(±34.51)	<0.001
**% of energy, mean (±SD)**	34.11 (±9.18)	33.90 (±9.17)	34.76 (±9.16)	<0.001
**Carbohydrate (g), mean (±SD)**	227.79 (±89.24)	228.70 (±90.01)	225.05 (±86.85)	0.145
**% of energy, mean (±SD)**	49.61 (±11.18)	50.11 (±11.28)	48.36 (±10.81)	<0.001

*P* value was calculated by Student’s t test for all continuous variables and chi-square test for all categorical variables. Abbreviation: BMI, body mass index; SD, standard deviation; PIR, poverty income ratio. *P* < 0.05 was considered statistically significant.

**Table 2:** Characteristics of the study population based on percent energy from carbohydrate quartiles.

Characteristic	Q1 (2.33, 42.44)	Q2 (42.44, 49.49)	Q3 (49.49, 56.65)	Q4 (56.65, 94.33)	*P*
**Number of participants**	1697	1698	1698	1698	
**Age, mean (SD)**	35.16 (±8.59)	34.66 (±8.64)	34.62 (±8.49)	35.05 (±8.55)	0.208
as categorical variable, n (%)					0.648
20–29	516(30.73)	547(32.21)	531(31.27)	502(29.56)	
30–39	583(34.72)	566(33.33)	585(34.45)	574(33.80)	
40–49	598(35.24)	585(34.45)	582(34.28)	622(36.63)	
**BMI, mean (SD)**	29.96 (±8.25)	30.10 (±8.14)	29.82 (±8.02)	29.38 (±7.72)	0.052
as categorical variable, n (%)					0.07
underweight	89(5.24)	96(5.65)	102(6.01)	125(7.36)	
normal weight	446(26.28)	426(25.09)	430(25.32)	409(24.09)	
overweight	436(25.69)	427(25.15)	445(26.21)	482(28.39)	
obese	726(42.78)	749(44.11)	721(42.46)	682(40.16)	
**Marital status, n (%)**					0.369
Single	684(40.31)	711(41.87)	663(39.05)	673(39.63)	
Married	1013(59.69)	987(58.13)	1035(60.95)	1025(60.37)	
**Race/ethnicity, n (%)**					< 0.001
Mexican American	247(14.56)	351(20.67)	340(20.02)	358(21.08)	
Other Hispanic	153(9.02)	163(9.60)	190(11.19)	180(10.60)	
Non-Hispanic white	688(40.54)	599(35.28)	589(34.69)	564(33.22)	
Non-Hispanic black	384(22.63)	366(21.55)	343(20.20)	331(19.49)	
Other	225(13.26)	219(12.90)	236(13.90)	265(15.61)	
**Educational level, n (%)**					< 0.001
Less than high school	261(15.38)	267(15.72)	331(19.49)	447(26.33)	
High school	330(19.45)	339(19.96)	342(20.14)	341(20.08)	
Above high school	1106(65.17)	1092(64.31)	1025(60.37)	910(53.59)	
**PIR, n (%)**					< 0.001
Low	632(37.24)	645(37.99)	713(41.99)	839(49.41)	
Middle	494(29.11)	553(32.57)	527(31.04)	492(28.98)	
High	571(33.65)	500(29.45)	458(26.98)	367(21.61)	
**Drinking status, n (%)**					< 0.001
Never	168(9.90)	228(13.43)	279(16.43)	375(22.08)	
Former	93(5.48)	112(6.60)	133(7.83)	161(9.48)	
Mild	458(26.99)	529(31.15)	529(31.15)	505(29.74)	
Moderate	506(29.82)	403(23.73)	364(21.44)	331(19.49)	
Heavy	472(27.81)	426(25.09)	393(23.14)	326(19.20)	
**Smoking status, n (%)**					0.003
Never	1149(67.71)	1215(71.55)	1218(71.73)	1235(72.73)	
Former	214(12.61)	176(10.37)	174(10.25)	142(8.36)	
Current	334(19.68)	307(18.08)	306(18.02)	321(18.90)	
**Diabetes mellitus, n (%)**					0.561
Yes	95(5.60)	109(6.42)	94(5.54)	108(6.36)	
No	1602(94.40)	1589(93.58)	1604(94.46)	1590(93.64)	
**Hypertension, n (%)**					0.656
Yes	251(14.79)	251(14.78)	248(14.61)	229(13.49)	
No	1446(85.21)	1447(85.22)	1450(85.39)	1469(86.51)	
**Hyperlipidemia, n (%)**					<0.001
Yes	1219(71.83)	1246(73.38)	1294(76.21)	1338(78.80)	
No	478(28.17)	452(26.62)	404(23.79)	360(21.20)	
**Physical activity, MET-min/week**	2166.00(846.00,5905.00)	1926.00(727.00,4986.00)	1927.00(7272.00,5105.00)	1926.00(726.00,5047.00)	0.038
**Total energy, mean (±SD)**	1866.56 (±653.83)	1937.98(±622.90)	1925.47 (±632.71)	1681.41 (±618.25)	< 0.001
**Protein (g), mean (±SD)**	82.15 (±33.17)	75.69 (±28.87)	69.95 (±27.20)	52.47 (±22.49)	< 0.001
% of energy, mean (±SD)	18.19 (±5.99)	15.91 (±4.44)	14.79 (±4.14)	12.73 (±3.93)	< 0.001
**Fat (g), mean (±SD)**	87.74 (±37.56)	80.25 (±30.35)	70.19 (±27.14)	47.69(±23.15)	0.000
% of energy, mean (±SD)	42.03 (±8.89)	36.97 (±6.03)	32.51 (±5.19)	24.94 (±6.09)	0.000
**Carbohydrate (g), mean (±SD)**	166.90 (±65.43)	223.44 (±72.60)	254.83 (±83.73)	265.94(±96.92)	< 0.001
% of energy, mean (±SD)	35.63 (±5.96)	46.11 (±2.05)	52.99 (±2.06)	63.72 (±6.18)	0.000

Analysis of One-Way ANOVA was used for continuous variables and Chi-square test was used for categorical variables. Abbreviation: BMI, body mass index; SD, standard deviation. PIR, poverty income ratio. *P* < 0.05 was considered statistically significant.

### 3.2 Association between the dietary carbohydrate intake and hyperlipidemia

Logistic regression analysis was used to examine the association between the percentage of energy from carbohydrate and the risk of hyperlipidemia, with the results presented in Table 3. A positive correlation between the percentage of energy from carbohydrate (both as a continuous and categorical variable) and the risk of hyperlipidemia was observed in all models, including both non-adjusted and adjusted models. After adjusting for all covariates, higher energy intake from carbohydrate was associated with an increased risk of hyperlipidemia (adjusted odds ratio (AOR): 1.014, 95% CI: 1.004–1.024). When analyzed as a categorical variable, the multivariable- AOR from the lowest to highest quintiles of percentage energy from carbohydrate were 1 (reference), 1.062 (95% CI: 0.892–1.264), 1.263 (95% CI: 1.031–1.546), and 1.411 (95% CI: 1.083–1.839), with a significant trend (*P* = 0.035).

**Table 3:** Association between percent energy from carbohydrate and hyperlipidemia among US reproductive-aged women in NHANES 2005–2020.

Carbohydrate intake as a percentage of energy in quartiles	Model 1	Model 2	Model 3
	OR (95%CI), *P*	AOR (95%CI), *P*	AOR (95%CI), *P*
Overall			
Q1	Reference	Reference	Reference
Q2	1.081(0.930, 1.257), 0.312	1.080(0.920,1.267), 0.346	1.062(0.892,1.264), 0.501
Q3	1.256(1.077, 1.265), 0.004	1.293(1.098,1.523), 0.002	1.263(1.031,1.546), <0.024
Q4	1.457(1.245, 1.706), <0.001	1.502(1.269,1.778), <0.001	1.411(1.083,1.839), <0.001
P for trend	<0.001	<0.001	0.035
Continuous scale (per SD increase)	1.014(1.009, 1.019), <0.001	1.015(1.009, 1.019), <0.001	1.014(1.004, 1.024), 0.007

Percentage of energy from carbohydrate quartiles range: Q1 = 2.33 to 42.44; Q2 = 42.44 to 49.49; Q3 = 49.49 to 56.65; Q4 = 56.65 to 94.33. Model 1: adjusted none; Model 2: adjusted for age, BMI, race, marital status, education level, PIR, drinking status, smoking status, diabetes mellitus, hypertension, and physical activity; Model 3: model 2+ total energy, percent energy from protein, and percent energy from fat. Abbreviation: OR, odds ratio; AOR, adjusted odds ratio; SD, standard deviation; *P* < 0.05 was considered statistically significant.

**Fig 2:**
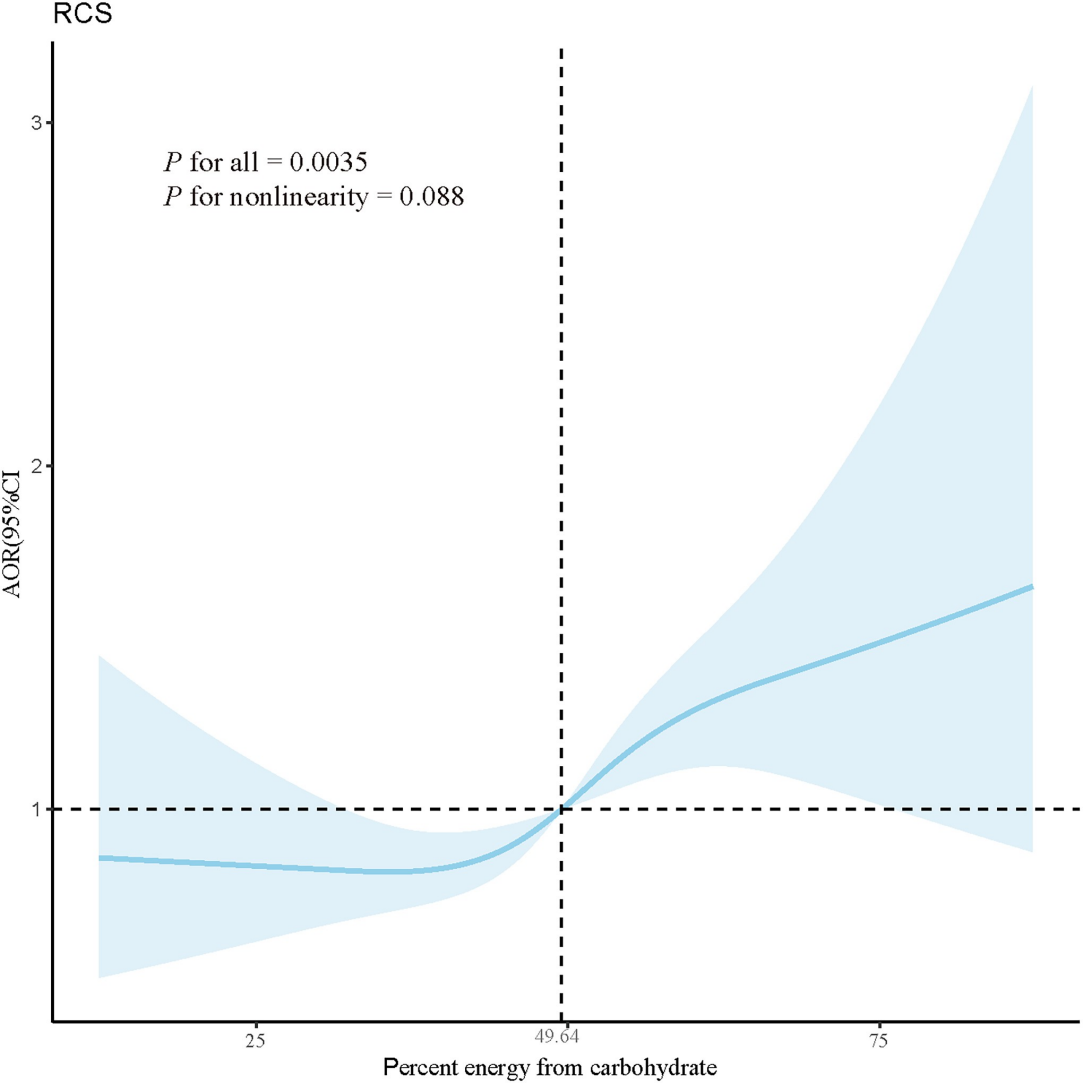
RCS depicts the dose-response relationship between percent energy from carbohydrate and hyperlipidemia. Note. The blue line represents AOR, and the blue transparent area represents 95% CI. AORs results are adjusted based on Model 3. Abbreviation: AOR, adjusted odds ratio; CI, confidence interval. *P* < 0.05 was set as the threshold of statistical significance.

As showed in Fig 2, a significant linear relationship was observed between the percentage of energy from carbohydrate and the risk of hyperlipidemia (*P* for nonlinearity = 0.088) in the RCS regression analysis. The curve in Fig 2 was divided into two sections by one inflection points at 49.64. When the percentage of energy from carbohydrate was below 49.64, there was an increase in the AOR, although the maximum value was less than 1. Conversely, when the percentage of energy from carbohydrate exceeded 49.64, the AOR >1 and increased sharply.

### 3.3 Subgroup and sensitivity analyses

**Fig 3:**
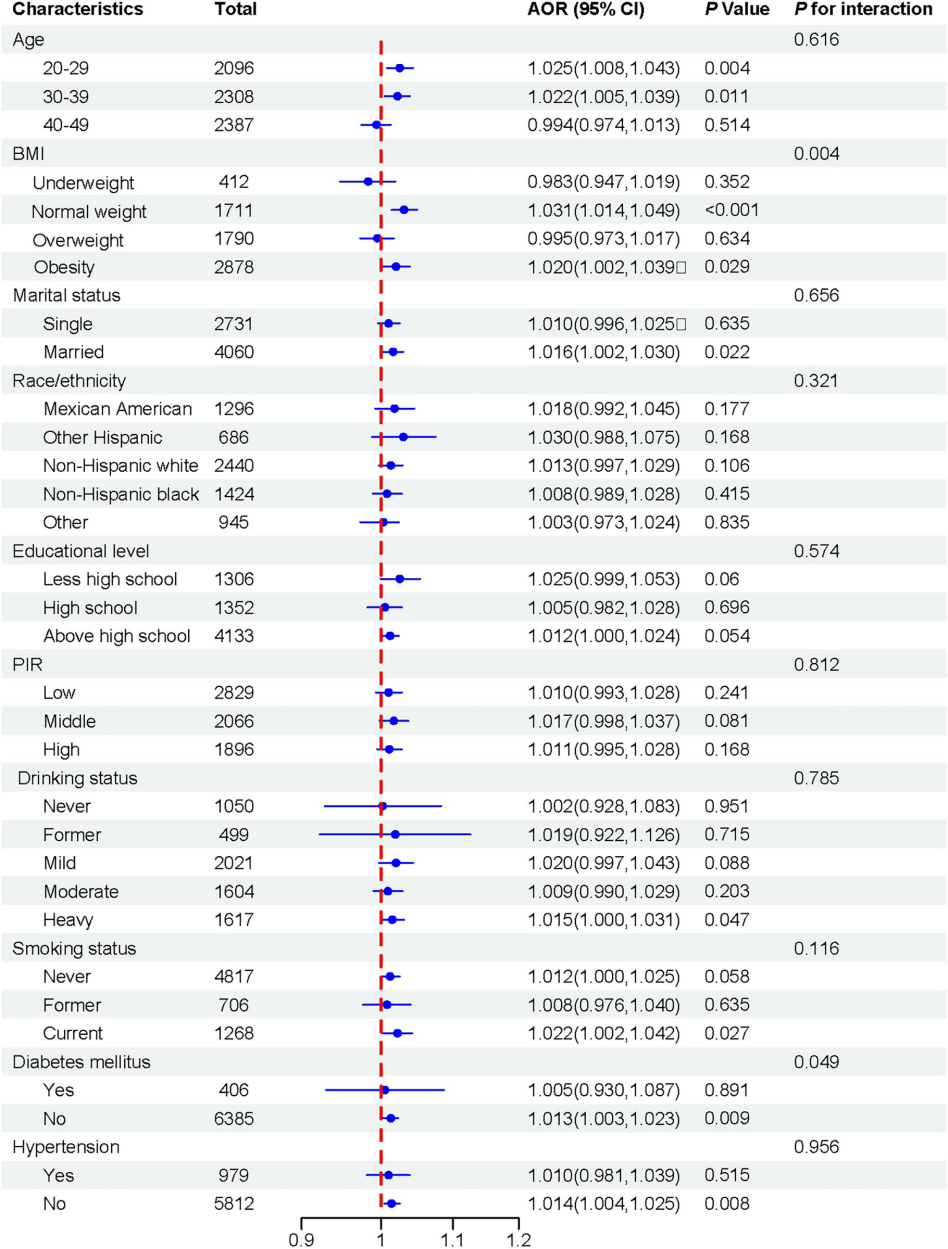
Subgroup analysis for the association between percent energy from carbohydrate and hyperlipidemia. Note. The outcome is adjusted for all covariables, with the exception of the corresponding stratification variable. Abbreviation: AOR, adjusted odds ratio; CI, confidence interval; BMI, body mass index; PIR, poverty income ratio. *P* < 0.05 was set as the threshold of statistical significance.

This study conduct subgroup analyses were stratified by gender and age, BMI, marital status, race, education levels, PIR, drinking status, smoking status, diabetes mellitus, and hypertension, as shown in Fig 3. The association between percentage of energy from carbohydrate and the risk of hyperlipidemia was stronger among participants aged 20–29 years (AOR: 1.025, 95% CI: 1.008–1.043) and 30–39 years (AOR: 1.022, 95% CI: 1.005–1.039). This association was also significant among women with normal weight (AOR: 1.031, 95% CI: 1.014–1.049), women with obesity (AOR: 1.020, 95% CI: 1.002–1.039), and married women (AOR: 1.016, 95% CI: 1.002–1.030). Furthermore, the association was observed among individuals with heavy drinking status (AOR: 1.015, 95% CI: 1.000–1.031), current smokers (AOR: 1.022, 95% CI: 1.002–1.042), and those without diabetes mellitus (AOR: 1.013, 95% CI: 1.003–1.023) or hypertension (AOR: 1.014, 95% CI: 1.004–1.025). Interaction tests indicated that factors had no significant impact on this positive association, except within the BMI and diabetes mellitus subgroup (*P* for interaction < 0.05).

This study also conducted a sensitivity analysis in which 406 individuals with diabetes mellitus were excluded (S1 Table). In the fully adjusted model, the AOR for hyperlipidemia across the quartiles of percentage energy from carbohydrate levels was 1.000 (reference) for Q1, 1.093 (95% CI: 0.915–1.305) for Q2, 1.277 (95% CI: 1.040–1.567) for Q3, and 1.445 (95% CI: 1.105–1.888) for Q4 (*P* for trend = 0.035). Furthermore, the percentage of energy from carbohydrate remained positively correlated with the risk of hyperlipidemia when analyzed as a continuous variable.

## 4. Discussion

This study found a positive association between the percentage of energy from carbohydrate and hyperlipidemia in logistic regression models, which remained robust after adjusting for all covariates. The RCS analysis showed a linear relationship between the percentage of energy from carbohydrate and the risk of hyperlipidemia. To our knowledge, this was the first cross-sectional study in the US to investigate the association between dietary carbohydrate intake and hyperlipidemia among reproductive-age women, using a large sample size.

Carbohydrate was considered the primary source of energy for the human body, providing the necessary fuel for various physiological processes and activities [34]. They were broken down by digestive enzymes into glucose, which was then absorbed by the cells of the intestinal wall and enters the bloodstream. Then, glucose was transported to various organs and tissues throughout the body for energy use [34]. Previous studies have reported significant associations between dietary carbohydrate intake and metabolic syndrome [35], coronary heart disease [35] and mortality [36, 37]. However, the association between carbohydrate intake and health outcomes was currently not fully understood. This study first transformed the percentage of energy from carbohydrate into categorical variables based on quartiles. It was found that compared to the population in Q1, individuals in Q3 and Q4 had a 26.3% and 41.1% increased risk of developing hyperlipidemia, respectively, indicating a significant trend. This result was further confirmed through RCS analysis based on logistic regression, demonstrating a linear positive correlation between the percentage of energy from carbohydrate and the risk of hyperlipidemia, with inflection points at 49.64. Although there were no studies exploring the relationship between a high-carbohydrate diet and the risk of hyperlipidemia, previous research reported its association with various adverse health outcomes. Park S-H et al. first reported an association between dietary carbohydrate intake and cardiovascular disease risk [38]. Their study, based on data from the third Korea National Health and Nutrition Examination Survey, showed that higher carbohydrate intake (70% of energy) was associated with increased BMI, blood pressure, fasting glucose, TG, and LDL levels in women [38]. Furthermore, one study using data from the US and Korean versions of the 2007–2012 NHANES found an association between high carbohydrate intake and metabolic abnormalities [19]. Their study revealed that higher carbohydrate intake was associated with lower HDL levels and higher TG levels in both US and Korean women. However, they did not explore the association between high dietary carbohydrate intake and hyperlipidemia. In this study, hyperlipidemia was diagnosed comprehensively, which was more comprehensive than studying individual indicators.

Several possible mechanisms could be involved in the association between a higher carbohydrate intake and hyperlipidemia. Excessive carbohydrate intake could lead to insulin resistance and abnormal glucose metabolism [39], affecting the regulation of blood lipid levels [40, 41]. A high-carbohydrate diet could also stimulate the liver to synthesize more triglycerides, resulting in elevated levels of triglycerides in the blood [42]. Moreover, some studies have demonstrated that a high-carbohydrate diet could potentially activate the fatty acid synthesis pathway, subsequently elevating blood lipid levels [43, 44].

When stratified by age, women aged 20–29 years or 30–39 years both exhibited an association between carbohydrate energy intake and hyperlipidemia. However, this association disappeared in women aged 40–49 years. It was widely knowledge that age was a risk factor of hyperlipidemia. With increasing age, changes occurred in body metabolism and hormone levels, which might have led to an imbalance in lipid metabolism [45], thereby increasing the risk of hyperlipidemia. However, the presence of hyperlipidemia in young adults might have indicated the existence of unhealthy dietary habits and lifestyles. It was very interesting to find that women with normal weight showed a stronger association between the dietary carbohydrate intake and hyperlipidemia. Individuals with normal weight typically had a balanced and healthy metabolism, making their bodies more susceptible to the effects of dietary carbohydrate intake. In addition, female who were married, had a heavy drinking states, or were current smokers showed a stronger association between the percentage of energy from carbohydrate and hyperlipidemia. Compared to single, a married relationship often tends to lead to healthier life choices, such as a regular and healthy diet, and positive emotions than single [46]. The risk of hyperlipidemia associated with the percentage of energy from carbohydrate may be further amplified in these women. Smoking damages vascular endothelial cells, promoting lipid deposition and thereby increasing the risk of elevated blood lipid levels [47]. Heavy drinking can disrupt lipid metabolism and affect the secretion of lipoproteins responsible for lipid transport, contributing to the development of hyperlipidemia [48]. The findings of this study suggest that for individuals who current smoke or engage in heavy drinking, a high-carbohydrate diet may further increase the risk of hyperlipidemia.

In subgroup analysis stratified by hypertension and diabetes mellitus, women without these conditions showed a stronger association between the percentage of energy from carbohydrate and the risk of hyperlipidemia. The absence of a correlation between dietary carbohydrate intake and hyperlipidemia could be due to the medications taken by patients with hypertension or diabetes mellitus, as well as intentional dietary modifications. In addition, no significant interactions were found between the percentage of energy from carbohydrate and other covariates, except for BMI and diabetes mellitus. This suggests that, besides BMI and diabetes mellitus, no other covariates were identified as influencing the association between dietary carbohydrate intake and the risk of hyperlipidemia.

### 4.1. Strengths and limitations

The strengths of this study as follows: Firstly, this was the first study to investigate the association between dietary carbohydrate intake and hyperlipidemia risk. Secondly, this study utilized a nationally representative sample of US adults and collected data using validated measures. However, this study also had several limitations. Firstly, dietary information was based on a single assessment at baseline, and participants might change their diets during the follow-up. Nonetheless, a single 24-hour recall could suffice given a sufficiently extensive sample size [38]. Secondly, this study was exclusively conducted within the US population, thus limiting the extrapolation of the results. Further research involving populations from other countries and regions was necessary to validate the finding of this study. Thirdly, the design of a cross-sectional study led to the inability of this study to determine any causality.

## 5. Conclusions

This study demonstrated a direct positive relationship between the dietary carbohydrate intake and the risk of hyperlipidemia among women of reproductive age. This finding will help to identify more effective dietary interventions to lower the incidence of hyperlipidemia among women of reproductive age.

## Supporting information

S1 Table Association between percent energy from carbohydrate and hyperlipidemia after excluding 406 individuals with diabetes mellitus. Note. Model 1: adjusted none; Model 2: adjusted for age, BMI, race, marital status, education level, PIR, drinking status, smoking status, diabetes mellitus, hypertension, and physical activity; Model 3: model 2+ total energy, percent energy from protein, and percent energy from fat. Abbreviation: OR, odds ratio; AOR, adjusted odds ratio; SD, standard deviation; P < 0.05 was considered statistically significant.
(PDF)
