# Supplementary material for: The association between dietary carbohydrate intake and the risk of hyperlipidemia among reproductive-aged women in the US: A cross-sectional study
Source: PLoS One. 2024 Oct 16;19(10):e0310184. doi: 10.1371/journal.pone.0310184 (PMC11482686; doi:10.1371/journal.pone.0310184)
Supplement: S1 Table — Note. Model 1: adjusted none; Model 2: adjusted for age, BMI, race, marital status, education level, PIR, drinking status, smoking status, diabetes mellitus, hypertension, and physical activity; Model 3: model 2+ total energy, percent energy from protein, and percent energy from fat. Abbreviation: OR, odds ratio; AOR, adjusted odds ratio; SD, standard deviation; P < 0.05 was considered statistically significant. (PDF) [file pone.0310184.s001.pdf]

Table S1 Association between percent energy from carbohydrate and hyperlipidemia after excluding 406 individuals with diabetes mellitus

**Carbohydrate intake as a percentage of energy in quartiles**	**Model 1**<br>**OR (95%CI), *P***	**Model 2**<br>**AOR (95%CI), *P***	**Model 3**<br>**AOR (95%CI), *P***
**Overall**			
Q1, (11.64, 42.40)	Reference	Reference	Reference
Q2, (42.40,49.52)	1.094(0.938,1.276),0.254	1.092(0.928,1.286),0.290	1.093(0.915,1.305),0.328
Q3, (49.52,56.71)	1.243(1.062,1.454),0.007	1.277(1.082,1.508),0.004	1.277(1.040,1.567),0.020
Q4, (56.71,90.61)	1.432(1.220,1.680), <0.001	1.490(1.255,1.768), <0.001	1.445(1.105,1.888),0.007
P for trend	<0.001	<0.001	0.035
**Continuous scale (per SD increase)**	1.013(1.008,1.018), <0.001	1.013(1.008,1.019), <0.001	1.013(1.003,1.023), 0.009

Model 1: adjusted none; Model 2: adjusted for age, BMI, race, marital status, education level, PIR, drinking status, smoking status, diabetes mellitus, hypertension, and physical activity; Model 3: model 2+ total energy, percent energy from protein, and percent energy from fat. Abbreviation: OR, odds ratio; AOR, adjusted odds ratio; SD, standard deviation; $P < 0.05$ was considered statistically significant.
